# COVID-19 vaccine hesitancy among pregnant women in an antenatal clinic in Durban, South Africa

**DOI:** 10.4102/sajid.v38i1.516

**Published:** 2023-08-31

**Authors:** Sahra Ashkir, Tashlen Abel, Olive P. Khaliq, Jagidesa Moodley

**Affiliations:** 1 Department of Obstetrics and Gynaecology, Faculty of Health Sciences, University of KwaZulu-Natal, Durban, South Africa; 2 Department of Paediatrics and Child Health, Faculty of Health Sciences, University of the Free State, Bloemfontein, South Africa

**Keywords:** pregnancy, vaccination, COVID-19, hesitancy, African ancestry

## Abstract

**Background:**

Mass administration of vaccines against severe acute respiratory syndrome coronavirus 2 (SARS-CoV-2) is the most efficient intervention against the coronavirus disease 2019 (COVID-19) pandemic. Recently, vaccinations were shown to be safe and effective during pregnancy. However, vaccination rates are low in low- and middle-income countries, and vaccine hesitancy is a major limiting factor.

**Objectives:**

To investigate the rate of COVID-19 vaccine hesitancy among pregnant women.

**Method:**

A cross-sectional questionnaire-based investigation of 313 unvaccinated pregnant women attending an antenatal clinic in Durban, South Africa (SA). The questionnaire included clinical and socio-demographic data, and reasons for vaccine hesitancy were recorded and evaluated.

**Results:**

Of 313 women participating, 126 (40.3%) were vaccinated against COVID-19, 21/313 = 6.7%; for those unvaccinated, 21/187 (13.9%) were planning to be vaccinated. However, most unvaccinated women, 174 of 187 (93%), showed COVID-19 vaccine hesitancy.

**Conclusion:**

The COVID-19 vaccination hesitancy among pregnant women in Durban, SA, is exceptionally high. This requires urgent attention by the relevant health authorities (both professional health organisations and the SA Department of Health) as many countries experience different waves of the variants of SARS-CoV-2 and herd immunity may not have been achieved.

**Contribution:**

This study showed a high vaccine acceptance hesitancy rate among pregnant women in SA.

## Introduction

The coronavirus disease 2019 (COVID-19) pandemic has resulted in over 6.5 million deaths worldwide, making it one of the deadliest in history.^1^ Vulnerable population groups such as those with underlying respiratory and cardiovascular disorders, the elderly and pregnant and lactating women are at particular risk of serious complications, hospitalisation and death.^1,2,3,4,5^ The known risk for pregnant women and their babies compared to the general population is well documented. Dashraath et al. reported higher hospital and intensive care unit (ICU) admissions in pregnant women compared to their nonpregnant controls.^6^ Viral infections can be more severe in pregnant women due to physiological changes during pregnancy. The immune system changes associated with pregnancy are directed towards foetal tolerance, while physiological changes are mainly in the respiratory and cardiovascular systems.^1,2,3,4^ It is well established that SARS-CoV-2 (severe acute respiratory syndrome coronavirus 2) targets cells in the respiratory tract and further affects other organs in the body. Moreover, both pregnancy and SARS-CoV-2 are procoagulants leading to increased thrombotic complications.^1,2,3,4,5,6^ Seroprevalence studies suggest that pregnant women in all trimesters are at an equal risk of contracting COVID-19 but that serious complications are higher in the third trimester leading to higher caesarean section rates and neonatal complications such as prematurity.^7^

Vaccination is the best way to protect pregnant women against COVID-19 complications.^1^ Recent evidence suggests that vaccination during pregnancy is safe for both women and their babies.^1^ Despite this, the initial phase 3 studies on COVID-19 vaccinations did not include pregnant women. Furthermore, given that in November 2022, the Omicron variant circulating in the United Kingdom (UK), the UK Health Security Agency recommends vaccination before a planned pregnancy, during pregnancy and during the lactating period.^1^ This is based on the fact that pregnant women who received two doses of an mRNA vaccine and a ‘booster’ are 88% less likely to be hospitalised than their unvaccinated counterparts.^1^ According to the COMIT (COVID-19 Maternal Immunization Tracker) recommendations, all pregnant and lactating women are advised to get vaccinated for COVID.^8^ It is therefore not surprising that other professional health bodies, such as the American College of the Obstetricians and Gynecologists and the Royal College of Obstetricians and Gynaecologists, advise pregnant women be offered COVID-19 vaccination during pregnancy and lactation.^1,2,3^ Despite COVID-19 vaccinations effectively preventing morbidity and mortality; vaccination rates, particularly in pregnancy, remain lower in sub-Saharan Africa than in other low- and middle-income countries (LMICs). This, in part, is attributed to ‘vaccine hesitancy’, mainly due to misinformation about vaccine origin, its safety and efficacy and fear of infertility.^9^ Vaccine hesitancy is a ‘delay in acceptance or refusal of vaccines despite availability of vaccination services’.^9^ The World Health Organization (WHO) has highlighted vaccine hesitancy as one of the top 10 threats to global health.^9^ Vaccine hesitancy is often fuelled by false information disseminated on social media. In addition, distrust towards the government and negative encounters with the local healthcare system ignite vaccine-hesitant attitudes.^10^

Studies regarding acceptance rates in pregnant women were conducted in several countries.^11,12,13,14^ A survey conducted in Singapore reported a 30% vaccine acceptance rate among pregnant women,^14^ while a similar study reported a 44% acceptance rate in the United States (US).^12^ Confidence in vaccination safety or efficacy, concern about COVID-19, belief in the relevance of vaccines to their own country, adherence to mask standards, faith in public health agencies and health research and views towards regular immunisations were the most significant predictors of vaccine uptake.^15,16^ The vaccine’s safety was also questioned because of false information on social media, which created fear in many individuals.^16^ COVID-19 vaccination was, however, shown to be safe in pregnant women in the year 2021. Pregnant women were given a choice to either accept or decline the vaccine. According to the American College of Obstetrics and Gynaecology, women were allowed to decline the vaccine if they had concerns about its safety and accept the vaccine after seeking advice from the healthcare professional.^16^

Current evidence from the UK shows that providing detailed information and addressing concerns of pregnant women regarding COVID-19 vaccination has led to a substantial uptake in vaccination numbers.^1^ This, however, may not be the case in LMICs. This study, therefore, aims to investigate vaccine hesitancy towards COVID-19 vaccination among pregnant women and their concerns about the COVID-19 vaccination in Durban, SA.

## Methods

### Study population

**FIGURE 1:**
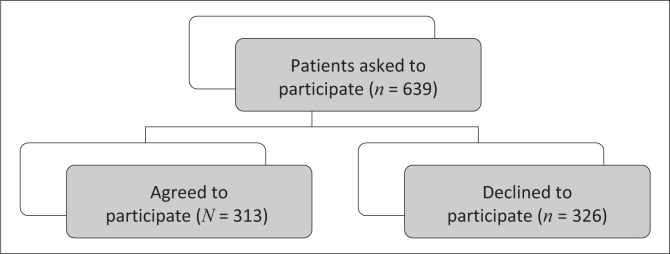
Diagrammatic representation of quantity of patients approached.

A precise, anonymous and non-judgemental survey was conducted at a Durban, SA, regional hospital based on an exclusion/inclusion criterion (Figure 1). Each participant was identified using a unique number system.

### Inclusion criteria

All pregnant women attending the antenatal clinic regardless of COVID-19 vaccination status or previous SARS-CoV-2 infection were eligible.

### Exclusion criteria

- Pregnant women who did not wish to participate in the study.

### Data collection

Multi-stage design sampling questionnaires were used to collect data from pregnant women who consented to participate. Questions relating to the demographic data, practices and attitudes of unvaccinated pregnant women were obtained. One of the researchers assisted consenting women who had difficulties interpreting the questionnaires. Patients visiting the antenatal clinic who consented to our study were given a questionnaire which was completed and collected on the same day. The collection process was ongoing between April 2022 and August 2022. At the beginning of April, the South African government lifted the national state of disaster and all COVID-19 lockdown regulations.^15^

### Data analysis

Statistical analysis was performed using GraphPad Prism software version 8.4.3 (GraphPad Software, San Diego, California, US). Vaccine acceptance rate comparisons were evaluated using Fisher’s exact test and presented as odds ratios (OR) and 95% confidence interval (CI). A *p*-value of < 0.05 was considered statistically significant for all tests. Data analysis was conducted using the Statistical Package for Social Sciences (SPSS) software (IBM Corp. Released 2020. IBM SPSS Statistics for Windows, Version 27.0, Armonk, New York: IBM Corp) to analyse the data. Descriptive statistics such as mean, standard deviation (s.d.) and range was used to summarise continuous data, while frequency counts and percentages were used to summarise categorical data. All parametric data were represented as mean ± s.d. while non-parametric data were presented as median and interquartile range.

### Ethical considerations

Ethical clearance to conduct this study was obtained from the University of KwaZulu-Natal Biomedical Research Ethics Committee (No. BREC/00003745/2022). Pregnant women, irrespective of their pregnancy trimester, were offered the opportunity to participate in the study, and informed written consent was obtained. Women were told that all the information would be kept confidential and that no names would be included in the questionnaire.

## Results

**FIGURE 2:**
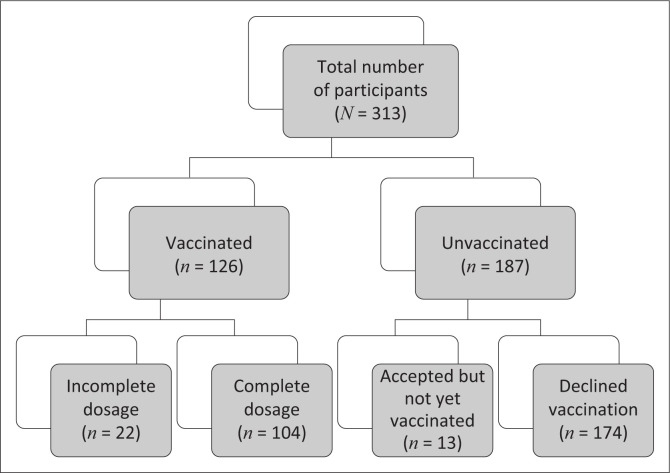
Study population groups.

Figure 2 graphically represents our study population groups and the number of participants in each group.

The study population consisted of 313 pregnant vaccinated and unvaccinated women. The patient demographics are summarised in Table 1. The mean maternal age of the population was 29 ± 6 years and mean gestational age was 27 ± 9 weeks. On average, all study participants lived in a household with 4 ± 2 members – with a median body mass index (BMI) of 31 kg/m^2^. Sixty pregnant women were HIV-positive, of whom 36 patients had CD4 cell counts available, averaging at 644 ± 200 cells/mm^3^ (Table 2). The mean duration of experiencing symptoms in days from SARS-CoV-2 infection was 7 ± 5 while the mean duration of symptoms after vaccination was 7 ± 13 (Table 3).

**TABLE 1:** Patient demographics (*n* = 313). All data were non-parametrically distributed.

Patient characteristic	*n*	Median	IQR (Q3–Q1)	Mean ± s.d.	Range
Maternal age (years)	313	29	34–25	29 ± 6	17–48
Vaccinated	126	31	34–26	30 ± 6	19–43
Unvaccinated	187	28	33–24	29 ± 6	17–48
Gestational age (weeks)	313	29	35–20	27 ± 9	5–41
Parity	313	1	2–0	1 ± 1	0–7
Gravidity	313	2	3–1	2 ± 1	1–8
Members in household	313	3	5–2	4 ± 2	1–27
Systolic BP (mmHg)	313	109	120–90	109 ± 14	74–165
Diastolic BP (mmHg)	313	66	77–57	66 ± 13	22–109
BMI (kg/m^2^)†	255	31	37–27	33 ± 10	17–113
Height (m)†	253	1.59	1.63–1.55	1.58 ± 0.1	1–1.76
Weight (kg)†	308	80	92–69	81 ± 19	45–150

BMI, body mass index; IQR, interquartile range; s.d., standard deviation; BP, blood pressure.

† , Data were not available for all patients.

**TABLE 2:** Other vaccinations, past and present pregnancy outcomes, co-morbidities. (*n* = 313).

Categories	Frequency	%
**Tetanus**		
Yes‡	80	63.0
**Influenza**		
Yes‡	54	43.0
**Previous pregnancy outcome**		
Alive	185	59.0
Previous pregnancy loss	24	8.0
Pregnant for the first time	104	33.0
**Medical history** †		
None	254	81.0
Diabetes	11	4.0
Chronic hypertension	5	2.0
Asthma	17	5.0
Cardiac conditions	1	0.3
Allergies	14	4.0
Anaemia	6	2.0
Tuberculosis	9	3.0
Skin cancer	1	0.3
Pulmonary embolism	1	0.3
Borderline personality disorder	1	0.3
**HIV status**		
Positive	60	19.0
Negative	253	81.0
**HIV treatment**		
Yes	59	19.0
**Flu-like symptoms during pregnancy**		
Yes	67	21.0
**COVID test result (*n* = 39)**		
Positive	6	2.0
Negative	29	9.0
Unavailable	4	1.0

† , Patients had more than one medical history;

‡ , Based on entire study population (*N* = 313).

Almost 60% (*n* = 185) of participants were discharged with live-babies in their previous pregnancy whilst 33% (*n* = 104) were pregnant for the first time (Table 2). More than 80% (*n* = 254) of the study population had no previous medical conditions. Only one HIV-positive women was not on antiretroviral therapy. Twenty-one percent (*n* = 67) experienced flu-like symptoms during pregnancy, 39 had COVID-19 tests performed and six tested positive. Two of the six tested positive for COVID-19 during their first trimester and experienced mild symptoms, while four experienced moderate to severe symptoms. Four of the six patients visited a general practitioner (GP) during their infection period and received symptomatic treatment.

Upon enquiring with patients, it was discovered that tetanus and influenza vaccines were administered to 45% (*n* = 142) and 31% (*n* = 97) of all participants, respectively. Participants were 20% less likely to accept the COVID-19 vaccine compared to the tetanus vaccine, OR (Cl 95%) = 0.8114 (0.5918–1.110), *p* = 0.2256. Whereas, participants were significantly (50%) more likely to accept the COVID-19 vaccine compared to the Influenza vaccine, OR (Cl 95%) = 1.500 (1.072–2.083), *p* = 0.0193. These vaccines were administered to pregnant women who received the COVID-19 vaccine and those who did not. Less than half of our participants already received the COVID-19 vaccine (40%; *n* = 126), of which 96 (31%) patients were vaccinated before pregnancy and 30 were vaccinated during pregnancy. Of the 30 participants who were vaccinated during pregnancy, 20 patients received their vaccine in the first trimester (Table 3).

**TABLE 3:** Descriptive statistics for the COVID-19 vaccinated group (*n* = 126).

Categories	Frequency	%†
**Vaccinated against COVID-19**		
Before pregnancy	96	76
During pregnancy	30	24
**Gestational age at vaccination**		
1st trimester	20	16
2nd trimester	9	7
3rd trimester	1	1
**Where were you told about the COVID-19 Vaccine** ‡		
Clinic	66	52
Other	52	41
**Type of symptoms after COVID-19 vaccination** §		
Dizziness	15	12
Weakness	8	6
Pain at the injection site	24	19
Other	33	26
**Type of COVID-19 vaccination** ¶		
Pfizer (2 doses)	54	43
J&J4 (1 dose)	59	47
Sinovac (2 doses)	1	1
Do not know	12	10
Completed dosage	104	83

Note: Duration of symptoms after vaccination (days): *n* = 50, Median = 3, Interquartile range (Q3-Q1) = 7–2, Mean ± s.d. = 7 ± 13, Range = 1–90.

COVID-19, coronavirus disease 2019; J&J, Johanson & Johanson.

† , Percentage of the total population (*N* = 313);

‡ , Patients were informed at more than one location;

§ , Certain patients experienced more than one symptom;

¶ , Johnsons and Johnsons.

Of the 126 vaccinated against COVID-19, 49 experienced complications after vaccination, with pain at the injection site (19%) being the most typical symptom. Following vaccination, 36% of the population consumed paracetamol. The Johnson & Johnson vaccine was the most commonly (47%) administered vaccine. Eighty-three percent of the population completed the COVID-19 vaccine dosage, while 17% (*n* = 22) were incomplete.

When the unvaccinated patients were asked if they would consider being vaccinated in the future, 93% (*n* = 174) of our study’s unvaccinated population (*n* = 187) said ‘no’ (Table 4). The commonest reason for declining the COVID-19 vaccination was disbelief in the vaccine’s efficacy at a rate of 57% (*n* = 106).

**TABLE 4:** Descriptive statistics for the COVID-19 non-vaccinated group (*n* = 187).

Categories	Frequency	%
**Did you know pregnant women can receive the COVID-19 vaccination?**		
Yes	122	65
**Would you like to be vaccinated against COVID-19?**		
Yes	13	7
No	174	93
**Concerns against COVID-19 vaccination** †		
Is it safe for my baby and myself?	179	96
Will it affect the baby during breastfeeding?	42	22
Potential to be infected even after vaccination	6	3
Not interested in COVID-19 and vaccinations/Do not believe in the disease	106	57
Personal choice/Religion	8	4
Waiting turn	3	2
Declined to answer	5	3
**Source of information about the COVID-19 vaccine**		
Social media	90	48
Family and friends	57	30
Newspaper	28	15
News television	79	42
Other	9	5
**Tetanus vaccine**		
Yes	62	33
**Influenza vaccine**		
Yes	43	23

† , Each patient had > 1 reason.

## Discussion

This study included 313 pregnant women, of which 187 were unvaccinated (Table 1 and Table 4). Hence, this latter number served as our population of interest. Based on these 187, a vaccine hesitancy rate of 93% was found (Table 4). However, the overall hesitancy in this study population was considerably lower (55%). In this study, the most common reason for hesitancy was concerns about the vaccine’s safety for the baby and mother (*n* = 179, 96%), and 42 women were worried about the baby’s safety during breastfeeding. These reasons are similar to a report in Italy where the hesitancy rate was 40% that mentioned that most women had concerns about vaccine safety during pregnancy.^17^ Another study in Iran reported a lower hesitancy rate (43%) to the COVID-19 vaccine compared to the current research. Two of the reported reasons for hesitancy were similar to our study, which included women’s concern about the safety of the vaccination for the unborn baby and disbelief in the vaccine’s efficacy. Another reason, not mentioned by our study population that resulted in vaccine hesitancy in Iran was the lack of information about the vaccine from healthcare professionals.^18^

Furthermore, this study reflected 20% less likelihood of accepting the COVID-19 vaccine compared to the tetanus vaccine; whereas pregnant women were significantly (50%) more likely to accept the COVID-19 vaccine compared to the influenza vaccine. This is an indication that the population was aware of the severity of COVID-19 infection compared to the common influenza infection. Alarmingly, only 45% and 31% of our population received the tetanus and influenza vaccines, respectively. Although all women are offered the routine vaccines, they are given the opportunity to decline vaccination. Improvement of the routine vaccine rates may be achieved through counselling pregnant women on the benefits of receiving and risks of not receiving the routine vaccines. Importantly, the studies discussed here have obtained and processed their data in various methods; some of which differ from the current study.

A study in Turkey that included only unvaccinated pregnant women investigated the factors relating to COVID-19 vaccination hesitancy and reported that 62.7% of the women chose not to vaccinate out of fear of side effects that could arise and cause them and their unborn babies harm.^19^ These reasons are similar to the current study’s; however, the hesitancy is lower in Turkey compared to our findings but slightly higher than other studies mentioned thus far.

Pregnant women in Cameroon had a lower hesitancy rate to the vaccine (46%) than our study. Moreover, the reasons for hesitating were similar to those found in the current study.^20^ Similar to the hesitancy rates in Cameroon, a study in the US also reported a hesitancy rate of 46% among pregnant women. Of the 46%, 37% were concerned about safety, 22% reported a lack of information and 15% reported disbelief in vaccine efficacy.^21^ A study in Romania reported a 52.2% hesitancy rate for different reasons, including not being fearful of getting infected with COVID-19, believing false rumours about the vaccine from social media, believing that COVID-19 is non-existent and not trusting that vaccinations, in general, are effective.^22^ Uganda reported a hesitancy rate of 58.6% in pregnant women.^23^ The reasons were also different from the reasons of the current study. These included the belief that COVID-19 is only fatal to individuals with comorbidities, the vaccine causes infertility and COVID-19 infection, misinformation on alcohol consumption during COVID-19 and lack of knowledge about the availability of the vaccine and vaccination sites.^23^

A study in Nigeria also reported a lower hesitancy rate (31.6%) than the current study. The commonest reason for hesitancy was concerns about vaccination safety and efficiency (30.9%).^24^ Another study in Africa was conducted in Ethiopia, where the COVID-19 vaccination hesitancy rate was reported as 68.7%. The majority that declined vaccination reported disbelief in the vaccine and its health benefits.^25^

The reasons reported in our study for declining vaccination are also supported by Neumann-Böhme et al.^26^ A disbelief in the vaccine and concerns about vaccination risks are usually due to a lack of trust in the government and healthcare system.^27^ Additionally, vaccine hesitancy is influenced by conspiracy theories, which increase during times of fear, uncertainty and insecurity, much like the situation created by the COVID-19 pandemic.^28^ Anti-vaccination conspiracy theories reduced vaccination intentions by causing feelings of powerlessness, disillusionment and mistrust in authorities.^29^ Thus, the Departments of Health must apply effective measures to reduce the impact of conspiracy theories, including addressing religious elements surrounding the views and increasing awareness of COVID-19 to reduce the stigma associated with COVID-19.^30^ Similar to Dodd et al., our results showed that patients who do not accept the vaccine were more likely to argue that the threat of COVID-19 and the danger of the infection were not legitimate.^31^

Of the unvaccinated population in this study, most women showing reluctance to vaccinate were unemployed (54%) compared to the unemployment rate of the vaccinated population (37%) (Table 5 and Table 6). This may be due to the requirement at some workplaces for everyone to be vaccinated to ensure the safety of others. Descriptive analysis of this study data showed that women with a tertiary education (42%) was less likely to decline the COVID-19 vaccine compared to women without a tertiary education (52%) (Tables 5 and 6). A study in France showed that more tertiary-level women accepted the COVID-19 vaccination than those without a tertiary-level education.^32^ Similarly, vaccination hesitancy in Uganda was higher among the less educated compared to the educated population.^23^

**TABLE 5:** Race, education, occupation and place of dwelling in the coronavirus disease 2019 unvaccinated (*n* = 187).

Socioeconomic status	Frequency	%
**Race**		
African ancestry	174	93
Mixed racial group	2	1
Indian people	9	5
White people	2	1
**Education**		
Primary	16	9
Secondary	92	49
Tertiary	79	42
**Occupation**		
Professionally skilled	13	7
Semi-skilled	45	24
Self-employed	9	5
Student	19	10
Unemployed	101	54
**Place of dwelling**		
Informal settlement	17	9
Urban (city)	117	63
Semi-urban (suburbs)	53	28

Note: Almost half (49%) of unvaccinated pregnant women had a secondary education, with most (54%) unemployed. Sixty-three percent of the unvaccinated women hailed from the city.

**TABLE 6:** Race, education, occupation and place of dwelling in the coronavirus disease 2019 vaccinated (*n* = 126).

Socioeconomic status	Frequency	%
**Race**		
African ancestry	91	72
Mixed racial group	6	5
Indian people	27	21
White people	2	1
**Education**		
Primary	3	2
Secondary	58	46
Tertiary	65	52
**Occupation**		
Professionally skilled	27	21
Semi-skilled	36	29
Self-employed	2	1
Student	15	12
Unemployed	46	37
**Place of dwelling**		
Informal settlement	9	7
Urban (city)	84	67
Semi-urban (suburbs)	33	27

Note: More than half (52%) of the vaccinated women held a tertiary education, albeit most were unemployed (37%). Sixty-seven percent of the vaccinated pregnant women were from the city.

Higher education is associated with vaccine acceptance; however, our study reflected the inverse (Table 5 and Table 6).^33^ A lack of acceptability among the higher educated may be associated with increased skepticism about COVID-19 information.^34^ Reportedly, it is less probable that urban dwellers will be willing to vaccinate against COVID-19^35,36^ although there are conflicting reports^22,37^. This is concerning as urban residents are largely educated with a significant influence on the population and social media platforms.^38^ Consequently, it may be possible that the dissemination of incorrect information is more prevalent among influential individuals; some may reflect the views of these individuals within the general population.^38^

This study also found that the younger population is more hesitant to vaccinate than the older population that is accordance with previous studies.^35,36,37^ It is plausible that the older participants are more attentive to the television and radio news, which are more reliable sources of information than social media.^36^ Several reports have indicated a negative association between social media use and vaccine acceptance,^22,38,39,40^ although others have reported a positive correlation.^41,42,43^ Additionally, the heightened hesitancy among younger women may be due to an invulnerability bias.^44^

Even though vaccination hesitancy is high in the pregnant population in SA, the numbers differ in the general population as reports show an acceptance rate of 81.6% in South Africa in a global survey,^45^ similar to reports from studies in Germany^46^ and China.^13^ Additionally, a study in Cape Town, SA conducted among healthcare workers reported a COVID-19 vaccine hesitancy rate of 41% due to a lack of trust in vaccine efficacy.^37^

### Strengths and limitations

According to the authors knowledge, this is the first study investigating vaccine hesitancy among pregnant women in Durban, South Africa. The population studied in South Africa experienced much inequity as in a LMIC. The current study included pregnant women of all age groups, those who regularly see medical providers, and those who do not, as well as different economic and educational backgrounds. The questionnaire was administered to patients in person to accommodate those who are not literate.

A limitation of our study was the inclusion of pregnant women from only one South African city. Including participants from other cities within the country may influence vaccine acceptance rates due to the different developmental rates of the cities. Our study included women who received the COVID-19 vaccine before pregnancy as well as during pregnancy. This could potentially skew the vaccine hesitancy curve as pregnant women are generally less inclined towards medicating and vaccinating during their pregnancy. Importantly, this study was conducted during a time when there was a low infection rate; the country lifted the state of disaster status and lockdown levels. A country in this state may make its citizens feel that COVID-19 and the vaccine are less important, ultimately increasing vaccine hesitancy among the population. A bias of our study is the selection of pregnant women from a regional (referral) hospital as these participants were already suspected to have or diagnosed with a pregnancy-related complication; hence, the results of this study cannot be generalised to the general pregnant population. Additionally, large number of pregnant women declined to participate in the study which may emphasise a high vaccine hesitancy rate. Less than half of this study population received the routine vaccines (tetanus and influenza); however, the study did not account for the reasons of not receiving these vaccines.

## Conclusion and recommendations

The COVID-19 vaccine hesitancy rate among pregnant South African women was substantially high compared to other African and non-African countries. The most commonly reported reason for vaccine hesitancy is concerns regarding the safety of the baby and mother. This should be addressed at a governmental and public heath level by increasing COVID-19 awareness. The spread of misinformation through social media should be controlled through increased cyber monitoring by social media platforms. Although only a few participants were concerned about the lack of research on the COVID-19 vaccine and pregnancy, it is paramount that we establish future investigations to identify the long-term effects of the vaccine in pregnant women and offspring. This study provides a platform for large-scale investigations to determine vaccine hesitancy and its associated risks among pregnant women with greater accuracy.

## References

[1] ACOG. COVID-19 vaccines and pregnancy: Key recommendations and Messaging for clinicians [homepage on the Internet]. 2022 [cited 2022 Dec 19]. Available from: https://www.acog.org/covid-19/covid-19-vaccines-and-pregnancy-conversation-guide-for-clinicians

[2] Cobb NL, Collier S, Attia EF, Augusto O, West TE, Wagenaar BH. Global influenza surveillance systems to detect the spread of influenza-negative influenza-like illness during the COVID-19 pandemic: Time series outlier analyses from 2015–2020. PLoS Med. 2022;19(7):e1004035. 10.1371/journal.pmed.1004035 35852993 PMC9295997

[3] World Health Organization. WHO coronavirus disease (COVID-19) dashboard [homepage on the Internet]. 2022 [cited 2022 Dec 29]. Available from: https://covid19.who.int

[4] Akhtar H, Patel C, Abuelgasim E, Harky A. COVID-19 (SARS-CoV-2) infection in pregnancy: A systematic review. Gynecol Obstet Invest. 2020;85(4):295–306. 10.1159/000509290 32728006 PMC7490507

[5] Phoswa WN, Khaliq OP. Is pregnancy a risk factor of COVID-19? Eur J Obstet Gynecol Reprod Biol. 2020;252:605–609. 10.1016/j.ejogrb.2020.06.058 32620513 PMC7320674

[6] Dashraath P, Nielsen-Saines K, Madhi SA, Baud D. COVID-19 vaccines and neglected pregnancy. Lancet. 2020;396(10252):e22. 10.1016/s0140-6736(20)31822-5 PMC7723327 32861313

[7] Crovetto F, Crispi F, Llurba E, Figueras F, Gómez-Roig MD, Gratacós E. Seroprevalence and presentation of SARS-CoV-2 in pregnancy. Lancet. 2020;396(10250):530–531. 10.1016/s0140-6736(20)31714-1 32771084 PMC7831869

[8] Berman Institute of Bioethics & Center for Immunization Research JHU. COVID-19 maternal immunization tracker (COMIT) [homepage on the Internet]. [cited 2023 Apr 25]. Available from: www.comitglobal.org

[9] World Health Organization. Ten threats to global health in 2019 [homepage on the Internet]. 2019 [cited 2022 Oct 02]. Available from: https://www.who.int/news-room/feature-stories/ten-threats-to-global-health-in-2019

[10] Ackah BBB, Woo M, Stallwood L, et al. COVID-19 vaccine hesitancy in Africa: A scoping review. Glob Health Res Policy. 2022;7(1):21. 10.1186/s41256-022-00255-1 35850783 PMC9294808

[11] Ellington S, Olson CK. Safety of mRNA COVID-19 vaccines during pregnancy. Lancet Infect Dis. 2022;22(11):1514–1515. 10.1016/s1473-3099(22)00443-1 35964615 PMC9371585

[12] Sutton D, D’Alton M, Zhang Y, et al. COVID-19 vaccine acceptance among pregnant, breastfeeding, and nonpregnant reproductive-aged women. Am J Obstet Gynecol. 2021;3(5):100403. 10.1016/j.ajogmf.2021.100403 PMC8146275 34048965

[13] Tao L, Wang R, Han N, et al. Acceptance of a COVID-19 vaccine and associated factors among pregnant women in China: A multi-center cross-sectional study based on health belief model. Hum Vaccin Immunother. 2021;17(8):2378–2388. 10.1080/21645515.2021.1892432 33989109 PMC8475603

[14] Jayagobi PA, Ong C, Thai YK, et al. Perceptions and acceptance of COVID-19 vaccine among pregnant and lactating women in Singapore: A cross-sectional study. MedRxiv. 2021:2021–06. 10.4103/singaporemedj.SMJ-2021-259 PMC11479004 37077051

[15] Skjefte M, Ngirbabul M, Akeju O, et al. COVID-19 vaccine acceptance among pregnant women and mothers of young children: Results of a survey in 16 countries. Eur J Epidemiol. 2021;36(2):197–211. 10.1007/s10654-021-00728-6 33649879 PMC7920402

[16] American College of Obstetricians Gynecologists. COVID-19 vaccination considerations for obstetric–gynecologic care [homepage on the Internet]. [Updated December 2021; cited 2022 Oct 13]. Available from: https://www.acog.org/clinical/clinical-guidance/practice-advisory/articles/2020/12/covid-19-vaccination-considerations-for-obstetric-gynecologic-care

[17] Bianchi FP, Stefanizzi P, Di Gioia MC, Brescia N, Lattanzio S, Tafuri S. COVID-19 vaccination hesitancy in pregnant and breastfeeding women and strategies to increase vaccination compliance: A systematic review and meta-analysis. Exp Rev Vac. 2022;21(10):1443–1454. 10.1080/14760584.2022.2100766 35818804

[18] Firouzbakht M, Sharif Nia H, Kazeminavaei F, Rashidian P. Hesitancy about COVID-19 vaccination among pregnant women: A cross-sectional study based on the health belief model. BMC Pregnancy Childbirth. 2022;22(1):1–9. 10.1186/s12884-022-04941-3 35918665 PMC9344440

[19] Sezerol MA, Davun S. COVID-19 vaccine hesitancy and related factors among unvaccinated pregnant women during the pandemic period in Turkey. Vaccines. 2023;11(1):132. 10.3390/vaccines11010132 36679977 PMC9863552

[20] Gunawardhana N, Baecher K, Boutwell A, et al. COVID-19 vaccine acceptance and perceived risk among pregnant and nonpregnant adults in Cameroon, Africa. PLoS One. 2022;17(9):e0274541. 10.1371/journal.pone.0274541 36099295 PMC9469991

[21] Kiefer MK, Mehl R, Costantine MM, et al. Characteristics and perceptions associated with COVID-19 vaccination hesitancy among pregnant and postpartum individuals: A cross-sectional study. BJOG. 2022;129(8):1342–1351. 10.1111/1471-0528.17110 35104382

[22] Citu IM, Citu C, Gorun F, et al. Determinants of COVID-19 vaccination hesitancy among romanian pregnant women. Vaccines. 2022;10(2):275. 10.3390/vaccines10020275 35214732 PMC8874778

[23] Kabagenyi A, Wasswa R, Nannyonga BK, et al. Factors associated with COVID-19 vaccine hesitancy in Uganda: A population-based cross-sectional survey. Int J Gen Med. 2022;15:6837–6847. 10.2147/ijgm.s372386 36061966 PMC9432568

[24] Ogbuabor DC, Chime AC. Prevalence and predictors of vaccine hesitancy among expectant mothers in Enugu metropolis, South-east Nigeria. J Public Health Policy. 2021;42(2):222–235. 10.1057/s41271-020-00273-8 33568746

[25] Hailemariam S, Mekonnen B, Shifera N, et al. Predictors of pregnant women’s intention to vaccinate against coronavirus disease 2019: A facility-based cross-sectional study in southwest Ethiopia. SAGE Open Med. 2021;9:20503121211038454. 10.1177/20503121211038454 34434555 PMC8381422

[26] Neumann-Böhme S, Varghese NE, Sabat I, et al. Once we have it, will we use it? A European survey on willingness to be vaccinated against COVID-19. Eur J Health Econ. 2020;21(7):977–982. 10.1007/s10198-020-01208-6 32591957 PMC7317261

[27] Jennings W, Stoker G, Bunting H, et al. Lack of trust, conspiracy beliefs, and social media use predict COVID-19 vaccine hesitancy. Vaccines. 2021;9(6):593. 10.3390/vaccines9060593 34204971 PMC8226842

[28] Douglas KM. COVID-19 conspiracy theories. GPIR. 2021;24(2):270–275. 10.1177/1368430220982068

[29] Jolley D, Douglas KM. The effects of anti-vaccine conspiracy theories on vaccination intentions. PLoS One. 2014;9(2):e89177. 10.1371/journal.pone.0089177 24586574 PMC3930676

[30] Khan YH, Mallhi TH, Alotaibi NH, et al. Threat of COVID-19 vaccine hesitancy in Pakistan: The need for measures to neutralize misleading narratives. Am J Trop Med Hyg. 2020;103(2):603–604. 10.4269/ajtmh.20-0654 32588810 PMC7410483

[31] Dodd RH, Cvejic E, Bonner C, Pickles K, McCaffery KJ. Willingness to vaccinate against COVID-19 in Australia. Lancet Infect Dis. 2021;21(3):318–319. 10.1016/s1473-3099(20)30559-4 PMC7326391 32619436

[32] Marić J, Gama-Araujo I. Implications of the COVID-19 pandemic in education and vaccine hesitancy among students: A cross-sectional analysis from France. Int J Logist Res Appl. 2022; 1–20. 10.1080/13675567.2022.2042225

[33] Solís Arce JS, Warren SS, Meriggi NF, et al. COVID-19 vaccine acceptance and hesitancy in low- and middle-income countries. Nat Med. 2021;27(8):1385–1394. 10.1038/s41591-021-01454-y 34272499 PMC8363502

[34] Cooper S, Van Rooyen H, Wiysonge CS. COVID-19 vaccine hesitancy in South Africa: How can we maximize uptake of COVID-19 vaccines? Expert Rev Vaccines. 2021;20(8):921–933. 10.1080/14760584.2021.1949291 34252336

[35] Tlale LB, Gabaitiri L, Totolo LK, et al. Acceptance rate and risk perception towards the COVID-19 vaccine in Botswana. PLoS One. 2022;17(2):e0263375. 10.1371/journal.pone.0263375 35120163 PMC8815939

[36] Gan L, Chen Y, Hu P, et al. Willingness to receive SARS-CoV-2 vaccination and associated factors among Chinese adults: A cross sectional survey. Int J Environ Res Public Health. 2021;18(4):1993. 10.3390/ijerph18041993 33670821 PMC7922368

[37] Wiysonge CS, Alobwede SM, De Marie CKP, et al. COVID-19 vaccine acceptance and hesitancy among healthcare workers in South Africa. Expert Rev Vaccines. 2022;21(4):549–559. 10.1080/14760584.2022.2023355 34990311

[38] Burger R, Köhler T, Golos AM, et al. Longitudinal changes in COVID-19 vaccination intent among South African adults: Evidence from the NIDS-CRAM panel survey, February to May 2021. BMC Public Health. 2022;22(1):422. 10.1186/s12889-022-12826-5 35236319 PMC8889513

[39] Ghaddar A, Khandaqji S, Awad Z, Kansoun R. Conspiracy beliefs and vaccination intent for COVID-19 in an infodemic. PLoS One. 2022;17(1):e0261559. 10.1371/journal.pone.0261559 35020721 PMC8754330

[40] Dambadarjaa D, Altankhuyag GE, Chandaga U, et al. Factors associated with COVID-19 vaccine hesitancy in Mongolia: A web-based cross-sectional survey. Int J Environ Res Pub Health. 2021;18(24):12903. 10.3390/ijerph182412903 34948511 PMC8701794

[41] Gewirtz-Meydan A, Mitchell K, Shlomo Y, Heller O, Grinstein-Weiss M. COVID-19 among youth in Israel: Correlates of decisions to vaccinate and reasons for Refusal. J Adolesc Health. 2022;70(3):396–402. 10.1016/j.jadohealth.2021.11.016 34952782 PMC8610826

[42] Ghaffari-Rafi A, Teehera KB, Higashihara TJ, et al. Variables associated with coronavirus disease 2019 vaccine hesitancy amongst patients with neurological disorders. Infect Dis Rep. 2021;13(3):763–810. 10.3390/idr13030072 34562997 PMC8482072

[43] Berenson AB, Chang M, Hirth JM, Kanukurthy M. Intent to get vaccinated against COVID-19 among reproductive-aged women in Texas. Hum Vaccin Immunother. 2021;17(9):2914–2918. 10.1080/21645515.2021.1918994 34081572 PMC8381790

[44] Choi JTS. The psychology of pandemics: Preparing for the next global outbreak of infectious disease. Asian Comm Res. 2020;17(2):98–103. 10.20879/acr.2020.17.2.98

[45] Lazarus JV, Ratzan SC, Palayew A, et al. A global survey of potential acceptance of a COVID-19 vaccine. Nat Med. 2021;27(2):225–228. 10.1038/s41591-020-1124-9 33082575 PMC7573523

[46] Führer A, Pacolli L, Yilmaz-Aslan Y, Brzoska P. COVID-19 vaccine acceptance and its determinants among migrants in Germany-results of a cross-sectional study. Vaccines. 2022;10(8):1350. 10.3390/vaccines10081350 36016238 PMC9413826

